# Video Conferencing Dysmorphia: Assessment of Pandemic-Related Body Dysmorphia and Implications for the Post-lockdown Era

**DOI:** 10.7759/cureus.22965

**Published:** 2022-03-08

**Authors:** Ashish Sarangi, Swarada Yadav, Jayasudha Gude, Wail Amor

**Affiliations:** 1 Geriatric Psychiatry, Baylor College of Medicine, Houston, USA; 2 Internal Medicine, Premier Medical Associates, The Villages, USA; 3 Psychiatry, Hackensack University Medical Center, Hackensack, USA; 4 Psychiatry, Texas Tech University Health Sciences Center, Lubbock, USA

**Keywords:** videoconference, obsessive-compulsive disorder, pandemic, body dysmorphia, zoom dysmorphia

## Abstract

Background: The coronavirus 2019 (COVID-19) pandemic has led to global effects on human interaction and mental health. The most drastic changes are seen in ways people continue to stay connected with each other. Video-conferencing applications like Zoom gained popularity and have become the primary means of communication for social or work events and meetings. These applications have also in many places replaced face-to-face healthcare visits and have penetrated school-based learning. The long-term implications of this digital technology on self-esteem and body image require further study.

Main Body: Video-conferencing applications have led to people being more conscious of their appearance and this has resulted in increased cases of body dysmorphic disorder (BDD). There is increased focus on body appearance and cosmetic procedures to fix minor defects. Although the treatment for BDD is like depression, it requires the personalization of therapy specific to the needs of the patient.

Conclusion: In this review, we aim to highlight the impact of the pandemic on body image and the long-term implications of virtual conferencing. The review also highlights available pharmacological and non-pharmacological treatment approaches in the management of body dysmorphic disorder related to virtual video conferencing.

## Introduction and background

Body dysmorphic disorder (BDD), also referred to sometimes as dysmorphophobia, is categorized as an obsessive-compulsive disorder (OCD) according to the Diagnostic and Statistical Manual of Mental Disorders (DSM-V). It is defined as an obsession over minor shortcomings which appear insignificant to others. It is associated with repetitive behaviors which involve either constant grooming or checking one's appearance more frequently than an average person. This compulsion leads to significant deterioration in the levels of functioning [1].

The coronavirus 2019 (COVID-19) pandemic has brought premature changes to the way people interact. Work from home (WFM) has skyrocketed during the time of the pandemic [2]. According to a study conducted by Erik Brynjolfsson in 2020, about 50% of the employees who were employed pre-COVID-19 are now working remotely. Amongst this 50%, more than a third were commuting to work prior to the pandemic [3]. The majority of interactions, which were earlier almost always in-person, are now through video conferencing applications like Zoom, Go-to-meeting, Skype, and so forth [1]. The most concerning arguably has been the penetration of these technologies being utilized for school-based learning involving children younger than 16 years when body image has not fully developed. As we aim to progress towards the end of the pandemic and attempt to resume operations to pre-pandemic normalcy, many still prefer to work remotely and find comfort in working from home [4]. A recent survey in 2020 stated that around 62% of Americans were working from home and that most of this proportion would prefer continuing this trend even after the pandemic is under control and operations are back to normal [5].

Video-conferencing applications are web-based applications that provide quality sharing of audio, video, media, screen share, etc. Due to the pandemic, people were required to use these applications for online lectures, webinars, conferences, court hearings, follow-up appointments with clinicians among other reasons [5,6]. These applications allow the user to change several settings on the screen like magnification, changing backgrounds and even using filters to modify one's physical appearance such as lip and eye color [7]. Compared to an in-person meeting, where individuals can only tend to their appearance before the meeting, virtual conferences allow the user to have constant access to the way they appear [8,9]. The camera draws the user’s attention to features he or she would not have normally noticed in an actual meeting. This creates room for some people to develop tendencies wherein they meticulously stare at the screen to tweak their posture, change their emotions, and do other minute things rather than concentrate on the meeting’s agenda [1,4,10]. The behavior of repeatedly critiquing oneself, which is understandably prevalent in the entertainment industry, is now making its way into the normal workforce population. Table 1 below demonstrates common areas of concern in patients with BDD and examples of subsequent procedures sought by patients. 

**Table 1:** Common interventions used for perceived defects in various body parts

Body Part	Intervention
Face	Botox
Hair	Hair Transplants
Teeth	Veneers
Lips	Lip augmentation
Nose	Rhinoplasty
Eyes	Blepharoplasty

“Zoom dysmorphia” is used as a collective term for all the internet-based video conferencing applications and their effect on people’s perception of the way they look on a screen. The COVID-19 pandemic may have shone a brighter light on Zoom dysmorphia, which has been prevalent since the pre-COVID-19 era [1,4].

Interestingly, the prevalence of BDD is much higher in non-psychiatric settings than in psychiatric ones. The premise behind this observation is that many patients with undiagnosed BDD are frequent follow-ups in dermatological clinics where they are seeking procedures done to enhance and fix their appearance. According to a systematic review, an estimated 1.9% of adults were diagnosed as having BDD in the local community, while this percentage increased to 5.8% to 7.4% in psychiatric settings and further increased to a range of 9.2% to 20.1% in various cosmetic and dermatological settings [11]. Even psychiatric clinicians have been unfortunately known to miss the condition of BDD. Only after patients are further screened for BDD in an organized fashion, it is observed that the majority of newly detected BDD cases had no mention of BDD even once, in their previous medical records. Four studies, two in psychiatric outpatient and two in psychiatric inpatient settings have demonstrated the frequency of missed diagnosis of BDD [12-15]. Another factor contributing to the under-reporting of BDD is the individual’s own reluctance to share information regarding their discomforting thoughts with their healthcare providers. A study conducted in a psychiatric in-patient ward conveys the same observation [16]. The study demonstrated that reasons for not talking about their symptoms included fear of judgment, not being aware that there are available treatments for BDD, embarrassment, feeling that their doctor may not understand their plight, and assuming that others did not have a similar predicament.

The rise of telemedicine during the pandemic has also resulted in increased use of video conferencing for physician and non-physician visits. Taking into consideration the continued future use of technology to supplement traditional “face-to-face” visits, it seems imperative to review the pros and cons and psychiatric implications of virtual connectivity in the current and post-pandemic era.

## Review

Methods

For the purposes of this narrative review, the authors decided to conduct a thorough database search to study the impact of video conferencing on body image. Data were explored on Google Scholar and PubMed using keywords “Dysmorphia”, “Zoom”, “Videoconferencing”, “COVID-19”. The inclusion criteria included all publications since December 2020, written in the English language, about the human species. We excluded commentaries, book chapters, letters to the editors and publications which were not reviewed in the systematic and traditional manner, experimental studies, as well as studies that did not meet the outcome of interest.

Our initial search led us to 66 articles which after a thorough abstract review led us to finalize 35 articles. We did not perform any systematic review of our article or quality assessment of the selected publications used as reference. A thorough abstract review was conducted to highlight articles of interest.

Discussion

Patients with BDD are persistently concerned about different features of their looks. Virtually any portion of the human body may be involved in BDD, but the more common parts are the ones that are most exposed on video such as the head and face, particularly the nose, skin, and hair [17]. Other parts of the body may also be involved such as the eyebrows and neck. Patients may feel vulnerable about sagging skins around their jowls which may lead to them seeking cosmetic interventions such as botox.

The rise of video applications that allow one to alter one’s image while on video has also resulted in heightened anxiety regarding one’s appearance with often frantic efforts to “fix” any assumed blemishes. Patients may exhibit compensatory behaviors such as wearing excessive make-up, dressing in expensive clothing, applying background filters, or wearing extravagant jewelry to mask perceived deficiencies on video. Changing hairstyles or colors on a regular basis for different meetings may also indicate underlying cognitive errors regarding one’s appearance. Being hyper-focused on camera angles or camera positions may also suggest psychopathology.

Over an entire lifetime, a patient with BDD may be preoccupied with an average of five to seven different parts of his body [18]. Patients may be concerned about the overall appearance of their bodies, being too small, or having a low muscle tone. This is specifically known as the muscular dysmorphia type of BDD [19]. Patients spending time in an inpatient psychiatric setting because of BDD spend anywhere between three to eight hours daily overly noticing their appearances, with around one-fourth of this population spending more than eight hours per day. This level of concern with one’s appearance is associated with unwanted and negative emotions like shame, depression, anxiety, and even disgust [20].

The person’s awareness regarding his preoccupations is often lacking. These beliefs may be of a delusional nature which is around 32% to 38% in BDD patients [21]. Delusions and their presence add to the severity of BDD. According to ICD-11, there are two subtypes of BDD in relation to delusional beliefs: BDD with good insight and BDD with absent or poor insight. The former is characterized by the person maintaining that his beliefs may be false and is open to accepting other explanations for his feelings. There will be occasions when he or she is anxious, that the person loses this insight. The latter subtype is characterized by the person maintaining that his beliefs are true most of the time and is unaccepting of alternate explanations for his beliefs. Anxiety levels do not affect insight in this subtype [22].

As a result of these uncomfortable thoughts, patients with BDD are bound to have impairments in one or more circles of their lives like academic, occupational, and social life [23]. They are also more likely to stay at home rather than participate in social or educational activities which would involve interactions with people. A meta-analysis observed that people with BDD are four times more likely to have thoughts of committing suicide and about 2.6 times more likely to act upon these thoughts compared to individuals without BDD [24]. Since anxiety, depression, and stress are related consequences of BDD, it is hypothesized that many patients have BDD along with these co-morbidities. Others include substance use disorder, obsessive-compulsive disorder, and social phobia [25]. It is possible that at the time of diagnosing these co-morbid disorders, BDD may be missed as the main underlying cause, leading to a situation wherein the patient may not receive the correct treatment due to a missed underlying diagnosis.

Evidence-based treatments

Patients with symptoms suggestive of BDD often present to either primary care offices or specialty clinics such as dermatology or plastic surgery. It is important to recognize patients presenting with perceived defects utilizing a thorough history, physical examination, and screening tools. The treatment for BDD has a similar framework to depression as it involves the use of selective serotonin reuptake inhibitor (SSRI) and cognitive behavioral therapy (CBT). 

CBT involves several components or aspects of BDD that need to be worked on [26]. It involves a multidisciplinary approach from the therapist, psychiatrist, and the patient to form a model of the patient’s specific BDD symptoms. This is followed by utilizing cognitive techniques to restructure the patient’s thought process. Some of the techniques include Socratic questioning, the downward arrow method, and helping the patients realize the pros and cons of their BDD-related beliefs. Identifying situations that the patients avoid and helping them come up with strategies to decrease these behaviors is another core aspect that allows both the therapist and the patients to keep tabs on the progress. BDD patients focus on a small portion of their bodies when looking at themselves in the mirror. CBT helps these patients appreciate their entire body rather than fixating on one part of the body. Using descriptive words rather than negative words to describe themselves is often utilized as an exercise for BDD patients. Once the patients begin to make progress in reducing negative thoughts, CBT encourages them to tackle their deeper belief systems of worthlessness or inferiority that they may possess and are guided to include features like intelligence, morals, and other talents as part of their opinion of themselves. Lastly, throughout the CBT sessions, the focus is given to strategies to prevent relapse. There are specific CBT modules for different subtypes of BDD, for example, muscle and weight-based modules are specifically used for patients who use excess steroids to build more muscle mass because of their perception that they are not sufficiently big and muscular. Cosmetic procedures, negative mood, and skin picking modules are some others used for patients who have a muscular focus component to their BDD.

The efficacy of CBT has been analyzed by several studies, but most of them did not conduct head-to-head trials comparing CBT with another treatment to provide a control [27-29]. In one study, 54 patients diagnosed with BDD were randomized to receive either CBT or no treatment [27]. The CBT group patients received eight group sessions, each lasting for two hours, wherein thought modification was encouraged. There was a reduction in BDD-related symptoms in 82% of patients receiving CBT and the benefit persisted in 77% of them at follow-up visits. This can be considered as evidence that suggests that the benefits of CBT may continue to be present even after its cessation. Another study showed the lasting effects of CBT after discontinuation [29].

In another study, 36 patients with BDD were randomized to receive CBT or a 12-week waitlist. Each patient in the CBT arm received individual CBT for a total of 22 sessions over the span of 24 weeks. While there was no difference in the two groups at the end of 12 weeks, a statistically significant difference was reported at the end of the 24 weeks. This difference was also present at further follow-up visits. A study that compared CBT to anxiety management (AM) in BDD patients provided interesting results [30]. It was a 12-week-long study, at the end of which it was seen that CBT was far more superior to AM in BDD patients. CBT also had better outcomes in BDD patients with delusions and depressive symptoms, showing that the efficacy of CBT is not affected by the status of the patient's insight about his or her diagnosis. Another study compared the efficacy of CBT provided by a therapist on the internet (BDD-NET) to that of general online supportive therapy [31]. It was concluded that BDD-NET was more effective than online supportive therapy with 56% responding to treatment in the BDD-NET group versus 13% in the online supportive therapy group.

Although not much has been said about the dosing of selective serotonin reuptake inhibitors (SSRI) in BDD patients, experts have suggested that higher doses of SSRI than those used in depression may be required to effectively treat BDD. Some individuals may even require supratherapeutic doses for maximal efficacy [23]. This may be related to a similar etiological mechanism to patients diagnosed with OCD.

Many studies have been conducted to test the effectiveness of antidepressants on BDD. A randomized controlled trial (RCT) demonstrated that the SSRI fluoxetine was significantly more useful than placebo in BDD patients [32]. Another RCT concluded that clomipramine performed better than desipramine (a non-SSRI antidepressant) in controlling symptoms of BDD and depression [33]. The decision to continue SSRI therapy after symptom relief in BDD patients was studied by yet another RCT, which concluded that the time taken to relapse was longer in patients who continued the SSRI for another six additional months compared to those who were administered placebo for the same six months [34].

A lot is yet to be uncovered about the treatment of BDD. There are no studies comparing CBT and SSRI to evaluate which modality provided greater symptom reduction for BDD patients. Another area of research would be to look for any additive effects CBT may provide to SSRI’s efficacy of treatment and vice versa. Data on neuromodulation techniques and efficacy for symptom reduction in patients with BDD is currently limited.

## Conclusions

Video-conferencing popularity has dramatically increased during the COVID-19 pandemic. Social distancing rules and a ban on travel have led people from all age groups to use video-conferencing apps for their day-to-day social activities like meetings, birthdays, exercises, and so on. These video-conferencing applications have aggravated body dysmorphic disorder with serious long-term concerns. Patients with pre-existing BDD are spending an excessive amount of time and money getting multiple treatments to change perceived defects. It is imperative to screen patients in both primary care offices as well as specialty care offices for symptoms suggestive of body dysmorphia. It is also imperative to have a continued discussion regarding the utility of teleconferencing and its impact on mental health in the post-pandemic era.

The mainstay of treatment is a combination of cognitive-behavioral therapy and antidepressant medications (SSRI). On an individual basis, it remains to be seen which one of these two modalities is more effective in treating BDD and further research needs to be conducted on neuromodulation and alternative treatment approaches that can hopefully provide a more robust solution.
